# Electrospun Poly(vinyl alcohol) Composites Containing pH-Fractionated Kraft Lignin: Structural Characterization, Biocompatibility, and Immunomodulation

**DOI:** 10.1021/acsomega.5c09388

**Published:** 2026-03-05

**Authors:** Chamberttan Souza Desidério, Hugo Felix Perini, Beatriz Sodré Matos, Antônio Aprigio da Silva Curvelo, Marcos Vinicius da Silva, Carlo José Freire Oliveira, Luís Carlos de Morais

**Affiliations:** † Laboratory of Immunology and Omic Sciences - Department of Microbiology, Immunology, and Parasitology, Institute of Biological and Natural Sciences, Federal University of Triângulo Mineiro − UFTM, Uberaba, MG 38025-350, Brazil; ‡ Parasitology and Molecular Biology Research Laboratory - Department of Microbiology, Immunology, and Parasitology, Institute of Biological and Natural Sciences, Federal University of Triângulo Mineiro − UFTM, Uberaba, MG 38025-350, Brazil; § Department of Physical Chemistry, Organic Physical Chemistry Group, São Carlos Chemistry Institute, University of São Paulo - USP, São Carlos, São Paulo 13560-970, Brazil; ∥ Polymer research and technological application laboratory − LaPPAT - Institute of Exact, Natural Sciences, and Education (ICENE), Federal University of Triângulo Mineiro − UFTM, Uberaba, MG 38025-350, Brazil

## Abstract

Lignin and its derivatives exhibit attractive physicochemical and biological properties, including antioxidant, antimicrobial, and immunomodulatory activities; however, the relationship between lignin fractionation conditions and these biological effects remains poorly understood. Filling this gap is fundamental for the advancement of lignin-based materials in biomedical applications. In this study, Kraft lignin was fractionated by precipitation at three pH values (2, 5, and 8) and incorporated into poly­(vinyl alcohol) (PVA) matrices, which were subsequently processed by electrospinning. The components and the resulting composites were systematically characterized in terms of molar mass distribution, chemical structure, surface morphology, and colloidal properties using GPC, FTIR, SEM, zeta potential, and particle size analyses. The results demonstrated that pH-controlled fractionation significantly altered the molecular characteristics of lignin, dispersion behavior, surface charge, and filament morphologies. Notably, the lignin fraction obtained at pH 5 exhibited a favorable balance between particle size and colloidal stability, 339 nm and −38 mV, respectively. Biological evaluation revealed that all composites were cytocompatible with Vero cells, showing no significant reduction (less than 10%) in cell viability after 24 h of exposure. In contrast, immunological assays using human peripheral blood mononuclear cells (PBMCs) revealed distinct pH-dependent immune responses. The composite containing lignin fractionated at pH 5 preserved cell viability and selectively increased TNF-α production (p-value = 0.0008) without affecting IL-10 levels (p-value >0.05), indicating a controlled pro-inflammatory response. Overall, these results demonstrate that pH-guided fractionation is an effective strategy for adjusting the physicochemical and immunological behavior of electrospun PVA/lignin composites and identify the PVA-lignin system fractionated at pH 5 as a promising platform for biomedical applications requiring immunological modulation.

## Graphical abstract

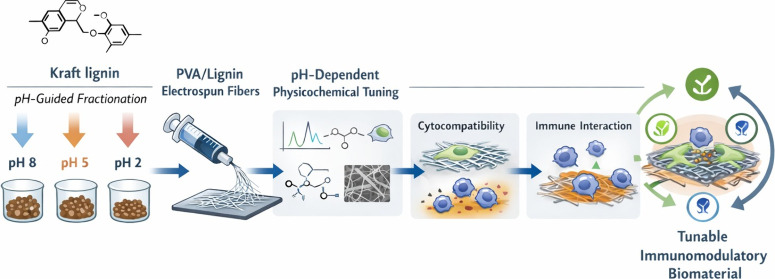

## 1 Introduction

Lignin (LIG) is an amorphous heteropolymer of organic origin, formed through the polymerization of three monolignol units: *p*-hydroxyphenyl (H), guaiacyl (G), and syringyl (S). These units are derived from *p*-coumaric, coniferyl, and sinapyl alcohols, respectively, and differ in the presence of methoxy groups in their structure.^1,2^ As an essential component of the cell wall in vascular plants, lignin provides structural rigidity, tissue impermeability, and pathogen resistance, playing a crucial role in plant adaptation to the terrestrial environment. Beyond its biological role, lignin has garnered increasing industrial interest due to its abundance in lignocellulosic biomass, a material mainly composed of cellulose, hemicellulose, and lignin.^3–5^ In 2023, the global lignin market was valued at USD 1.08 billion, with a projected compound annual growth rate (CAGR) of 4.5% between 2024 and 2030. This growth is primarily driven by the use of lignin in animal feed formulations and natural product applications.^6^

The versatility of lignin, attributed to its complex chemical structure, enables its application across multiple sectors, including the chemical, energy, and biomedical industries. In addition to its use in the production of biofuels and sustainable polymers, lignin has been investigated for the development of functional nanomaterials, particularly for controlled drug delivery and other biomedical applications. For instance, lignin-containing biocomposites have been employed in packaging, automotive materials, and biomedical devices. Moreover, its intrinsic antioxidant, antimicrobial, and ultraviolet radiation-absorbing properties further expand its potential for advanced biomaterial applications.^5,7–10^

However, lignin utilization is strongly dependent on its source, the chemical modifications applied, and the resulting physicochemical characteristics. Indeed, the incorporation of lignin into biocomposites presents technical challenges, including structural heterogeneity arising from its botanical origin and extraction methods, which may affect material uniformity, structural organization, and functional performance. Consequently, current research efforts focus on optimizing lignin fractionation, processing, and modification strategies to broaden its applicability and improve its technological properties.^11–13^ In the context of health-related applications, lignin has been successfully incorporated into electrospun poly­(vinyl alcohol) (PVA) matrices^14,15^ and other polymeric systems.^16,17^ Although these studies have yielded promising results, comprehensive evaluations using human biological samples remain limited, particularly regarding cytocompatibility, structural integration, and immunomodulatory effects.

In this context, we hypothesized that fractionation of Kraft lignin under controlled pH conditions would generate chemically distinct fractions capable of modulating cellular and immune responses when incorporated into electrospun PVA matrices. To test this hypothesis, Kraft lignin was fractionated at three different pH levels and subsequently integrated into PVA dispersions via electrospinning. The main findings demonstrate that lignin fractionation significantly influences material composition, cytotoxicity profiles, and immunomodulatory behavior, revealing a pH-dependent effect of fractionated lignin on cellular compatibility and immune response modulation. These results highlight the potential of pH-fractionated Kraft lignin as a tunable component for the development of electrospun biomaterials with tailored biological and immunological properties.

## 2 Methodology

### 2.1 Desugared Kraft Lignin

The method was adapted according to the method described by Feng and Wergener (1984).^1^ Approximately 5.0000 g of Kraft lignin was weighed and placed in a 1000 mL flat-bottomed flask with 800 mL of 2% (w/w) H_2_SO_4_ solution and left to reflux under stirring for 4 h. After this time, the sample was filtered through M-type porous plate filters. The filtrates were then washed until the pH remained close to neutrality. The samples were placed in an oven and left at the temperature of 105 ± 2 °C for 4 h, then placed in a desiccator for later use.

### 2.2 Fractionation of Lignin

Approximately 10 g of desugared Kraft Lignin (KL) was initially dissolved in a sodium hydroxide solution with a pH of 14. Hydrochloric acid (1 mol/L) was added slowly until the pH reached 8. After this adjustment, the solid fraction was separated from the liquid medium. More hydrochloric acid was then added to the liquid fraction until the pH dropped to 5, at which point the solid fraction was removed once again. Finally, the liquid fraction was acidified to a pH of 2, and the solid fraction was separated once more. After this step, all the solid fractions were dialyzed until the pH of the medium was close to neutrality. These samples were dried in an air circulation oven at 60 °C for 3 days. After this step, they were stored in desiccators containing silica gel. These fractionations give the samples KL-pH 2, KL-pH 5, and KL-pH 8.

### 2.3 Electrospun PVA with Fractioned Lignin

Electrospinning was carried out using an Instrutemp ITHY 60 kV Hipot source, with the negative terminal connected to a grounded metal collector and the positive terminal connected to the needle of a syringe containing the aqueous PVA dispersion. The syringe was mounted on an SDA 1800 syringe pump set to a flow rate of 1.0 mL·h^–1^. The distance between the needle tip and the collector was maintained at 10 cm. A voltage of 18 kV was applied under a relative humidity of 52%.

After preparing mixtures of aqueous PVA with 4% in weight of fractioned lignins, the dispersions were placed in 10 mL syringes containing a metal needle with an internal diameter of 45 μm and coupled to an electrospinning system. The syringe with the dispersion was coupled to an infusion pump, and the injection speed was adjusted to 1 mL min^–1^. After spinning, the material was dried and subsequently cross-linked with 750 μL of 50% glutaraldehyde (GTA) and 250 μL of concentrated HCl in isopropyl alcohol at 50 °C. The samples were washed with approximately 3 L of deionized water and then dried in an air circulation oven at 60 °C for 3 days. The nanocomposite electrospun samples were identified as PVA-KL-pH 2, PVA-KL-pH 5, and PVA-KL-pH 8.

### 2.4 Gel Permeation Chromatography (GPC)

Prior to GPC analysis, enhanced solubilization in THF was necessary. Approximately 1.0 g of lignin was acetylated using 20 mL of a solution of acetic anhydride and pyridine in a 1:1 volume ratio, placed in a 125 mL Erlenmeyer flask. The reaction was conducted at room temperature overnight, with continuous stirring at 200 rpm using a NT-375 shaker equipped with temperature control. After the reaction period, approximately 125 mL of ethanol was added while stirring. After 30 min, the mixture was evaporated using a rotary evaporator with a thermostated bath. Multiple additions and extractions with ethanol were performed until all traces of pyridine and acetic anhydride were removed. Finally, the acetylated samples were dried overnight at 50 °C, followed by further drying at 80 °C for 4 h, and then dried at 105 °C for 2 h.

The molar mass distributions of the fractionated lignins were determined using a SHIMADZU chromatographic system with tetrahydrofuran (THF) as the solvent. The data obtained were processed using GPC software for the CLASS-LC10 system. The following conditions were employed: Detector: Differential Refractive Index (model RID 6A) and a UV detector set to 254 nm. Column Setup: A PL-Gel precolumn followed by three columns in series: PLgel 500, 103, and 104 A from Polymer Laboratories. Filling Material: Poly­(styrene/divinylphenylbenzene) gel (PS/DVB). Eluent: THF.

### 2.5 Infrared (FTIR) in ATR Mode

FTIR measurements in ATR were performed on a compact FTIR spectrophotometer model ALPHA II (Bruker Corporation, Massachusetts, United States) in the range of 4000 to 400 cm^−1^, 32 scans, and a resolution of 2 cm^−1^. The technique allowed information on chemical groups of molecular structures to be obtained and thus correlated these data with those obtained by other analyses.

### 2.6 Particle Dispersion Characterization

The mean particle size (by DLS) and electrophoretic mobility (zeta potential) of the poly­(vinyl alcohol), fractioned Lignins at pH 2, pH 5, and pH 8 in aqueous media, and a mixture of these lignins, PVA in the RPMI culture medium, were obtained using a Malvern Zetasizer Nano-ZS90 Instrument (UK). Measurements were repeated three times for each sample to check the reproducibility.

### 2.7 Scanning Electron Microscopy (SEM)

The samples were placed on a support with carbon tape and then coated with metallic gold on an SCD050 (Leica) sputtering device. Next, the samples were analyzed by scanning electron microscopy EVO MA 10 (Carl Zeiss).

### 2.8 Cell Culture Conditions

The Vero cell line (VERO ATCC CCL-81, Manassas, VA, USA) was used to assess cytotoxicity. Cells were maintained in Roswell Park Memorial Institute −1640 (RPMI-Gibco Life Technologies, Paisley, UK) medium with 2 mM l-glutamine (Gibco Life Technologies, Paisley, UK), supplemented with 20 μg/mL of Gentamicin (Hytamicina – Brasil), and 10% fetal bovine serum (FBS- Gibco Life Technologies, Paisley, UK). For the tests, cells were plated in 96- and 24-well plates, in final volumes of 0.2 and 0.5 mL, respectively. The plates were incubated at 37 °C, with 5% CO2.

### 2.9 Cytotoxicity Assays

Cytotoxicity assays were performed by the resazurin assay method.^18^ Vero cells were detached using 0.25% trypsin, adjusted to a concentration of 5 × 10^5^ cells/mL, and seeded in 96-well culture plates containing the different lignin–PVA nanocomposite formulations. The plates were incubated at 37 °C under 5% CO_2_ for 24 h to allow cell attachment and interaction with the materials. After cell adhesion, the different nanocomposite formulations were tested: PVA, PVA+ (LIGNIN pH 2, LIGNIN pH 5, LIGNIN pH 8), with treatments performed in triplicate. Cells were exposed to the materials for 24 h. Subsequently, 5 μL of resazurin solution (2.5 mg/mL) was added to each well, and the plates were incubated for an additional 3 h at 37 °C under 5% CO_2_. Absorbance was measured at 570 and 600 nm^19^ using a spectrofluorometer (Bio Tek – ION). Data were expressed in percentage of viability in comparison to nontreated conditions. All essays were performed in triplicate and in three independent experiments.

Cytotoxicity (%) was calculated as follows:

$$\mathrm{Cytotoxicity}\;\left(\%\right)=\frac{\mathrm{Abs}\left(t\right)}{\mathrm{Abs}\left(c\right)}\times 100$$

where Abs (t) is the absorbance of cells treated with any compounds, and Abs (c) is the absorbance of cells not treated with compounds.

### 2.10 PBMC Culture and Quantification of Cytokines by ELISA

Peripheral blood mononuclear cells (PBMCs) were isolated by density gradient separation (PHARMACIA, SWEDEN). In summary, freshly collected blood was layered on 15 mL of Ficoll-Paque in 50 mL tubes (Thermo Fisher Scientific, Waltham, MA, USA). Afterward, centrifugation at 400g for 30 min at 20 °C without acceleration was applied, and the ″buffy coats″ were collected, pooled, resuspended in RPMI-1640, and centrifuged at 300g for 5 min at 20 °C with acceleration. The pellets were resuspended and centrifuged again. The collected PBMCs were resuspended, cell counts were determined using a Neubauer counting chamber, to obtain a final concentration of 10^6^/mL, and then cultured for 24 h in 24-well culture plates in a humidified atmosphere (5% CO_2_, 37 °C) under the following conditions: untreated PBMCs, electrospun PVA, electrospun nanocomposites PVA containing (LIGNIN pH 2, LIGNIN pH 5, LIGNIN pH 8) incubated at 37 °C, 5% CO_2_ for 24 h. After incubation, the supernatant was collected for cytokine measurement. Cytokine concentrations (TNF-α, IL-1β, and IL-10) were determined by ELISA (BD ELISA OptEIA kits) following the manufacturer’s specified assay recommendations. Triplicate samples were run, and results were equalized by comparison with standard curves expressed in pg/mL. The measurements were taken using the EnSpire Multimode Plate Reader spectrophotometer at a wavelength of 450–570 nm, in accordance with the manufacturer’s instructions. The PBMCs used were derived from cells designated for disposal from healthy individuals enrolled in studies approved by the ethics committee of the Federal University of the Triângulo Mineiro (UFTM) under protocols numbered 852 and 1475.

### 2.11 Statistical Analysis

Normal distribution and homogeneous variance were tested for all study variables. Then, the D’Agostino–Pearson test was used to assess normality. In cases of non-Gaussian distribution, the nonparametric Mann–Whitney test was applied. Multiple comparisons regarding median values for more than two groups were performed using the nonparametric Kruskal–Wallis test followed by Dunn’s test. Differences were considered statistically significant when the probability of their occurrence was less than *p* < 0.05 (5%). Statistical analysis was performed using GraphPad Prism software (GraphPad Software 8.0, La Jolla, CA, USA). To achieve the correlation analysis after finding the non-Gaussian distribution, Spearman’s correlation analysis was applied, where the correlation was considered significant when *p* < 0.05.

## 3 Results and Discussion

### 3.1 pH-Dependent Surface Morphology of Electrospun PVA/Lignin Filaments

Scanning electron microscopy (SEM) was used to examine the surface morphology of electrospun poly­(vinyl alcohol) (PVA) fibers containing lignin fractions obtained at different pH values. The SEM micrographs shown in Figure 1 (A–F) demonstrate that the pH employed during lignin fractionation significantly influences particle size, surface roughness, and the degree of PVA filament exposure, indicating a strong structure-processing relationship. At pH 2 (Figure 1 A–B), the lignin fraction formed a dense and relatively uniform coating on the PVA fibers, with limited filament exposure. The smooth surface morphology suggests the presence of finely dispersed lignin particles, consistent with increased lignin solubility and more homogeneous deposition under acidic conditions during fractionation, consistent with the data discussed later from the GPC results.

**Figure 1:**
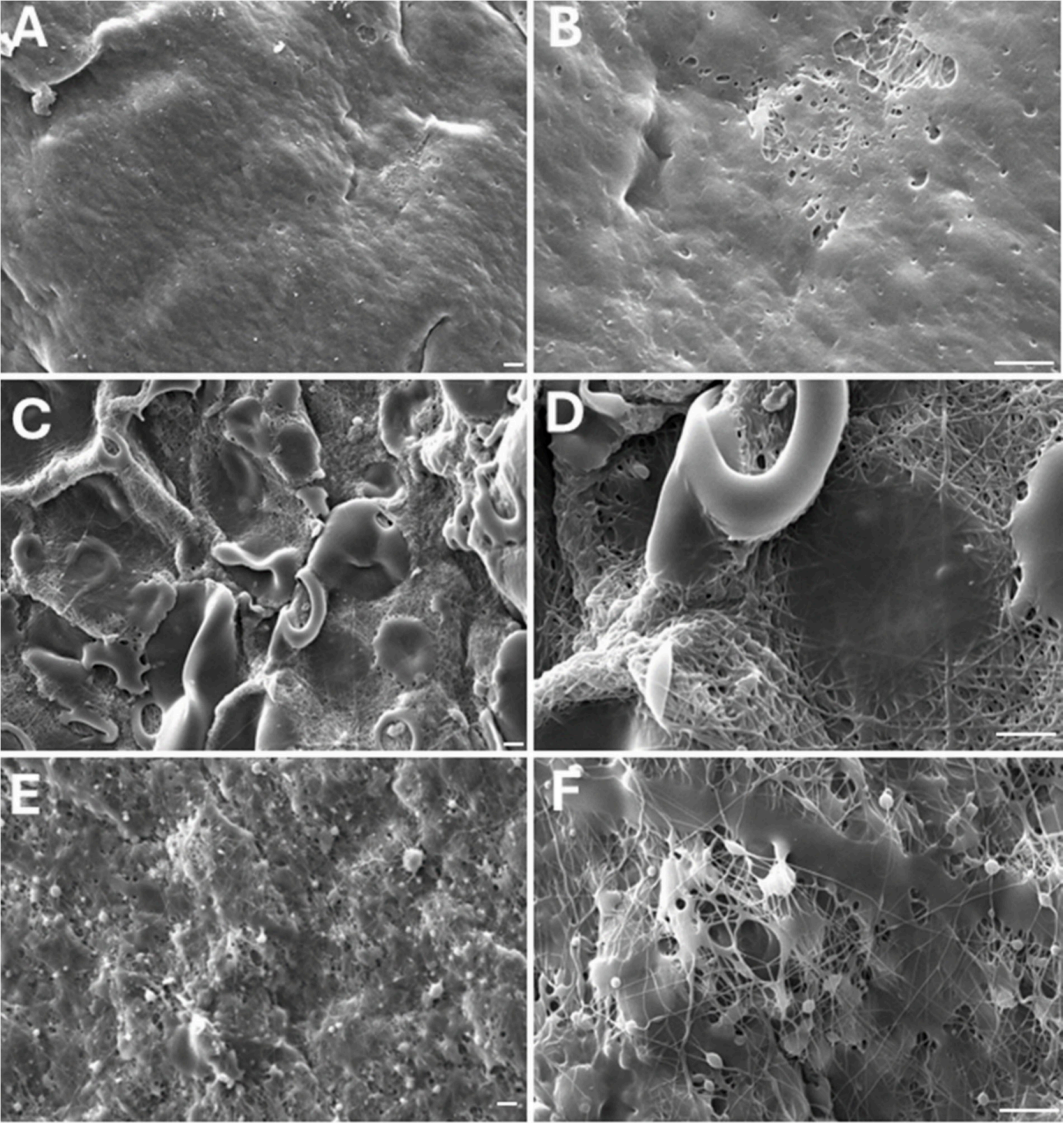
SEM images of samples containing PVA matrix with fractionated Kraft lignin, with magnifications of 1000x and 3500x, respectively. SEM images of samples containing PVA matrix with fractionated Kraft lignin. PVA-KL-pH 2 (A); PVA-KL-pH 2 (B); PVA-KL-pH 5 (C); PVA-KL-pH 5 (D); PVA-KL-pH 8 (E); PVA-KL-pH 8 (F). White bars = 10 μm. Images with magnifications of 1000x (A, C, E) and 3000x (B, D, F).

For samples prepared with lignin fractionated at pH 5 (Figure 1 C,D), larger and more heterogeneous particles were observed on the fiber surface, leading to increased roughness and partial exposure of the PVA filaments. This morphology indicates reduced coating continuity, likely associated with lignin aggregation at intermediate pH, which affects its dispersion during electrospinning. In contrast, fibers incorporating lignin fractionated at pH 8 (Figure 1 E,F) exhibited smaller particles than those observed at pH 5, resulting in a porous and highly irregular surface. Although PVA filaments remained visible, the increased surface porosity suggests altered lignin–polymer interactions under electrospun conditions, which may enhance surface area and accessibility.

These pH-dependent morphological differences are expected to influence subsequent biological responses and were therefore considered in the analysis of cytotoxicity and immunomodulatory assays using VERO cells. Similar surface characteristics have been reported for electrospun PVA systems incorporating water-soluble or alkaline lignin derivatives, where improved dispersion led to smoother and more uniform fiber morphologies.^20,21^

### 3.2 Molar Mass Distribution of Fractionated Kraft Lignin

Gel permeation chromatography (GPC) was employed to evaluate the molar mass distribution of the lignin fractions. The molar mass measurements obtained by GPC are presented in Table 1. The data indicate that decreasing pH levels are associated with an increased prevalence of lignin fractions with lower molar mass. Similar trends have been reported in GPC studies of lignins derived from hardwood and softwood sources.^22^ This similarity concerning values was found by Pinto and collaborators (2002).^23^ Additionally, investigations involving lignin fractionation using organic solvents have reported comparable values of Mw, Mn, and polydispersity.^24,25^ Higher polydispersity index (PDI) values suggest that, at lower pH levels, the disparity between Mn and Mw becomes more pronounced. This difference directly influences the content of aliphatic and aromatic hydroxyl (OH) groups,^22^ which can influence the production of electrospun composites of PVA and fractionated Kraft lignins.

**Table 1:** GPC of KL and Fractioned KL at Different pH’s^a^

Sample	Mn	Mw	*M*_w_/*M*_n_
KL-pH 2	190	2,791	14.71
KL-pH 5	564	3,059	5.43
KL-pH 8	335	3,396	10.13

a Mw: weight-average; Mn: number-average, and *M*_w_/*M*_n_: molecular mass polydispersity (PDI).

### 3.3 Chemical Structure of Fractionated Kraft Lignin by FTIR

To better understand the chemical structures of the fractionated lignins, FTIR-ATR analyses were performed, and the spectra are shown below in Figure 2. The FTIR spectra allowed us to highlight the signals related to the vibration modes of the chemical groups present in the Kraft lignin and in the fractions obtained from it at different pHs.

**Figure 2:**
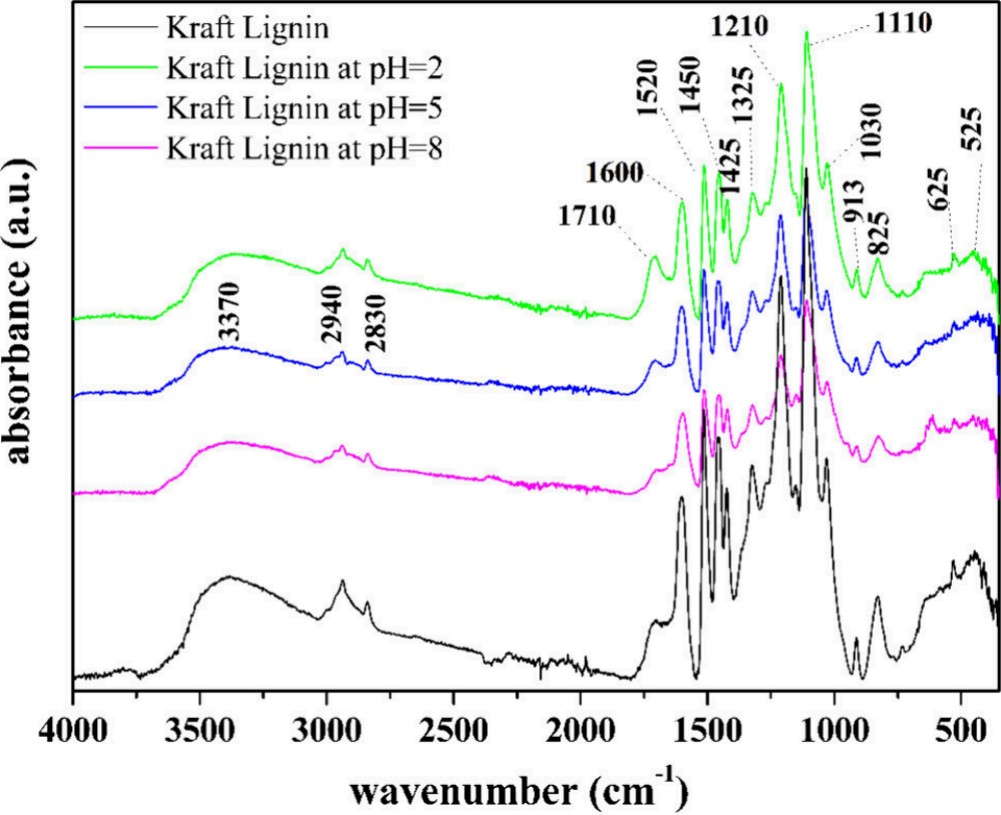
FTIR of Kraft lignin and fractioned lignins at pH 2, pH 5, and pH 8.

At 3370 cm^–1^, the band is attributed to OH groups derived from aromatic and aliphatic groups. The bands at 2940 and 2840 cm^–1^ belong to C–H asymmetric, and symmetric stretching from CH_2_ and CH_3_ groups, respectively. The signal at 1710 cm^–1^ belongs to conjugated carbonyl and carboxylic groups. Aromatic skeletal vibration signals appear in the range of 1600 to 1500 cm-1, typical of the lignin structure. The signal at 1450 cm^–1^ refers to the C–H bending vibration of methyl and methylene groups. The small signal seen at 1325 cm^–1^ can be attributed to C–O stretching from Syringyl plus Guaiacyl condensate rings.^26–28^ A strong vibration is seen at 1210 cm^–1^ associated with the overlapping C–C plus C–O plus C=O stretching signal. The signal at 1110 cm^–1^ belongs to various vibration modes attributed to C=O and in-plane deformations of aromatic −H (syringyl) and secondary alcohols. The in-plane aromatic C–H deformation occurs at 1110 cm^–1^ and the out-of-plane aromatic deformation occurs at 825 cm^–1^.^29^ The signal at 1030 cm^–1^ can be attributed to the overlap of the C–O, C–C stretching and C–OH bending signals in polysaccharides. Centered at 913 cm^–1^, the out-of-plane C–H deformations appear in aromatics.^30^ At 825 cm^–1^, the signal is the out-of-plane C–H vibration at positions 2, 5, and 6 of the Guaiacyl units. Finally, at 625 cm^–1^, out-of-plane C–H deformations occur at positions 2 and 6 (Syringyl) and 6 (Guaiacyl).

When multiple groups within the same region exhibit similar vibrational energy modes, their signals can interfere with each other. This interference can lead to band broadening and can even shift the peak position of the signal. Furthermore, in FTIR spectroscopy, absorbance values are influenced by the sample concentration and the molar absorptivities of the respective groups. To mitigate these issues, we can compare the peak values of two signals within the spectrum, allowing for a more accurate comparison of different spectra. Therefore, to better understand the information contained in the spectra in Figure 2, the data presented in Table 2 have been provided.

**Table 2:** Relationship of the Main Absorbances between Chemical Groups Obtained by FTIR from Kraft Lignin (KL), and Fractioned KL at pH 2, pH 5, and pH 8^a^

	absorbance ratio			
Signals (cm^–1^)	KL	Fractioned KL-pH 2	Fractioned KL-pH 5	Fractioned KL-pH 8
Hydroxyl	3387 (4.9)	3360 (4.2)	3404 (4.4)	3385 (3.6)
Carbonyl and Carboxylic	1700 (8.2)	1715 (4.5)	1706 (6.0)	1696 (7.3)
Aromatic skeletal	1600 (2.8)	1600 (2.4)	1602 (2.4)	1597 (2.4)
Aromatic skeletal	1514 (1.9)	1514 (1.9)	1514 (1.7)	1514 (1.8)
C–H of methyl and methylene groups	1456 (2.1)	1454 (1.9)	1454 (1.8)	1457 (1.9)
Syringyl plus Guaiacyl condensed rings	1325 (2.4)	1323 (2.2)	1323 (2.0)	1324 (2.2)
C–C plus C–O plus C=O stretching	1211 (1.3)	1210 (1.2)	1212 (1.2)	1211 (1.4)
C=O and deformations in the plane of aromatic C–H (syringyl) and the secondary alcohols	1109 (1.0)	1108 (1.0)	1112 (1.0)	1108 (1.0)
C–O, C–C stretching and C–OH bending in polysaccharides	1030 (2.3)	1029 (1.8)	1030 (2.0)	1029 (1.7)

a The values inside the parentheses express the division of the values at the maximum absorbances in relation to the reference value at 1109 cm^–1^ (normalized).

Comparisons should be made using the values from the KL sample. The reference signal was selected at 1109 cm^–1^ because it showed the highest absorbance. By dividing this reference value by the other absorbance values, we can obtain whole numbers, which will simplify the comparison between the spectra. The data presented are directly linked to this work’s proposal, as the values shown in the Table indicate the structural chemical changes in the lignin samples following fractionation by pH.

The analysis of band position changes highlighted the importance of hydroxyl and carbonyl/carboxylic groups. For the hydroxyl groups, the band in KL was observed at 3387 cm^–1^, which shifted to 3360 cm^–1^ for Fractioned KL at pH 2, and to 3404 cm^–1^ for Fractioned KL at pH 5. In terms of carbonyl/carboxylic groups, the most notable change occurred in Fractioned KL at pH 2, where the band shifted from 1700 cm^–1^ in KL to 1715 cm^–1^. These position changes are directly associated with energy changes in the vibration modes of these chemical groups.

The absorbance ratio between the bands showed significant differences in hydroxyl and carbonyl/carboxylic groups. For KL, the ratio was 4.9, indicating that the absorbance value of A1109 is 4.9 times greater than that of A3387. Furthermore, the lignins fractionated from KL at pH levels of 2, 5, and 8 exhibited ratios of 4.2, 4.4, and 3.6, which correspond to changes of 14%, 10%, and 17%, respectively.

Initially, it is important to note that the spectra shown in Figure 3A, corresponding to PVA samples with and without cross-linking, exhibit signal positions that may vary depending on the type of PVA used, namely, the degree of hydrolysis, as well described in the work of Mansur et al. (2008).^31^ The blue-highlighted regions correspond to the spectral domains where structural transformations in PVA are most responsive after chemical cross-linking. It can be observed that both before and after cross-linking with GTA, the PVA still displays OH stretching bands around 3310 cm^–1^, indicating that although some hydroxyl groups remain unreacted, the amount of cross-linking agent was sufficient to render the matrix water insoluble. The insolubility of the composite is a key characteristic for targeted applications.

**Figure 3:**
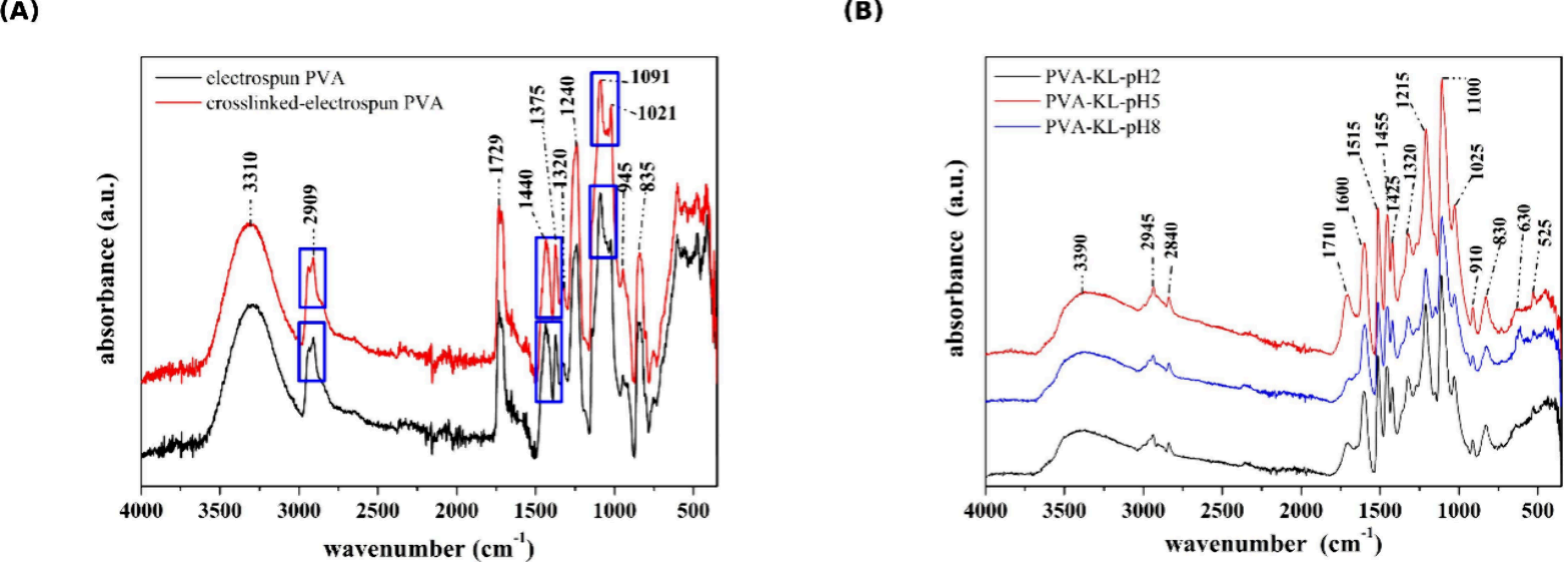
FTIR of electrospun PVA, and cross-linked electrospun PVA (A). Cross-linked electrospun PVA nanocomposites containing fractioned KL at pH 2, pH 5, and pH 8 (B).

Rezaee and Moghbeli (2014) also investigated electrospun PVA with and without cross-linking, in a manner similar to the present study.^32^ Their findings showed that when an excess of GTA is used, the OH band at approximately 3310 cm^–1^ disappears entirely. The signal at 2909 cm^–1^ is characteristic of C–H stretching in alkyl groups. Signals at 1729 cm^–1^ and 835 cm^–1^ are associated with the stretching of residual C=O and C–O groups, originating from the hydrolysis process of poly­(vinyl acetate), from which PVA is derived.

The bands at 1375 cm^–1^ and 1021 cm^–1^ correspond to C–OH stretching modes. In this study, these signals suggest that incorporation of GTA fragments into the PVA structure alters the vibrational profile, as also reported by Shaikh et al. (2012).^33^ This observation raises concerns, as GTA is known to exhibit a certain degree of toxicity. Subsequent results with cells presented later in this study may indicate whether residual GTA negatively impacted biological performance.

The region between 1150 cm^–1^ and 1085 cm^–1^ shows a stretching band attributed to C–O–C groups, which may originate from both cross-linking reactions and acetyl residues present in the PVA. Blue rectangles in Figure 3-A highlight the main spectral differences between electrospun PVA before and after GTA cross-linking. Notable changes include the appearance of a double C–H signal at 2909 cm^–1^ and the increase in intensity of the 1021 cm^–1^ band, which becomes more prominent after cross-linking. Similar spectral features have been reported in previous studies.^31,33^

As shown in Figure 3B, the FTIR-ATR spectra of the electrospun PVA composites with fractionated lignins exhibit the same characteristic absorption bands discussed in Figures 2 and 3A, suggesting no further significant structural modifications.

### 3.4 pH-Dependent Surface Charge, Colloidal Stability, and Interactions in Aqueous and Biological Media

An important parameter of this work was the measurement of Zeta Potential, which helps us understand the variations in positive and negative charges within the chemical structures of PVA, fractionated Kraft Lignin at different pH levels, as well as the culture medium (RPMI). It is worth noting that, since it was not possible to perform these analyses using nanocomposites, pure PVA was dissolved in water, and the results obtained are shown in Table 3. Similarly, PVA dissolved in the presence of RPMI and fractionated lignins at different pHs was analyzed to understand how each component affects the charge distributions on the particle surfaces. This is an important variable for the objectives that this work aims to investigate. The measurements indicate whether interactions between these components under controlled scientific conditions are advantageous or not. The results from Zeta Potential are shown in Table 3.

**Table 3:** Zeta Potential (ZP) of Poly­(vinyl alcohol), Fractioned Lignins at pH 2, pH 5, and pH 8 in Aqueous Media, and a Mixture of These Lignins, PVA in the RPMI Culture Medium, and Size by DLS

Sample in water	Measurements (mV) average	Size (nm)
KL-pH 2	–7.816 ± 1.475	842 ± 74
KL-pH 5	–38.146 ± 0.715	339 ± 14
KL-pH 8	–46.700 ± 1.042	536 ± 47
PVA	–20.193 ± 3.160	186 ± 69
PVA+ KL-pH 2	–3.424 ± 0.434	-----
PVA+ KL-pH 5	–3.899 ± 0.596	-----
PVA+ KL-pH 8	–7.524 ± 0.084	-----
PVA+RPMI	–7.606 ± 0.648	-----
KL-pH 2+RPMI	–12.325 ± 1.349	-----
KL-pH 5+RPMI	–14.645 ± 0.568	-----
KL-pH 8+RPMI	–14.636 ± 0.286	-----
PVA+KL-pH 2+RPMI	–12.657 ± 0.425	-----
PVA+KL-pH 5+RPMI	–3.899 ± 0.596	-----
PVA+KL-pH 8+RPMI	–7.524 ± 0.084	-----

The DLS data revealed that the sizes of Kraft lignins varied depending on the fractionation pH. It has been suggested that aggregation tends to occur when the Zeta Potential (ZP) falls between −30 and +30 mV.^34^ For the expected results, it is important to note that charge variations are not observed as a function of pH changes. Instead, three lignin fractions were obtained at different pH levels. The result of KL-pH 2, which had a ZP of −7.816 + 1.475 mV, is indicative of aggregation caused by the charges at the surface of the chemical structure. As indicated by the GPC data, this sample exhibited the smallest value of Mw in comparison to KL at pH 5 and pH 8; however, it displayed the highest value to particle size, which was 842 + 74 nm.

In contrast, samples KL-pH 5 and KL-pH 8 exhibited ZP values of −38.146 + 0.715 mV and −46.700 + 1.042 mV, both below −30 mV, respectively, which is associated with particle stability. Interestingly, the ZP values increased with increasing pH for KL. They also presented sizes of 339 + 14 and 536 + 47 nm, respectively. Another work also evaluated the ZP value at pH 5 and found a value similar to that found in our work.^35^ In this case, size here is more associated with the effect of how they were fractionated than the agglomeration caused by their ZP values, because based on the ZP values, the KL-pH 5 sample was expected to have a larger size due to its lower stability compared to the KL-pH 8 sample.

These ZP values can be explained by two main points. One is the fractionation process used in this work, which is a relation between pH and the KL structure. During the fractionation process, when pH 5 and pH 8 were reached, their respective solid fractions were collected, dialyzed, and dried, which contributed to the salt form prevailing, increasing the negative charges at structure surfaces. The other point is the presence of acidic groups on the KL structure; the ionization of the acid groups also contributes to the rise of the negatively charged surface.^36^

PVA ZP value solubilized in water showed a ZP value of −20.193 + 3.16 mV, similar to that found by Wiśniewska (2011), and showed a size of 186 + 69 nm.^37^ When we examine the ZP values of KL at pH 5 (−38.146 ± 0.715 mV), KL at pH 8 (−46.700 ± 1.042 mV), and PVA (−20.193 ± 3.16 mV), along with the new values obtained after mixing these KLs with PVA, it becomes evident that the ZP of PVA promotes interaction with the particles. This is because the ZP of PVA falls within the instability range, which is defined as values inside the range of +30 mV. It is important to note that samples KL-pH 5 and KL-pH 8 have a ZP of less than −30 mV, indicating they are stable particles. Meanwhile, PVA has a ZP of −20.193 mV. Despite these negative charges, their interaction was enhanced. This can be explained from a physical-chemical perspective by considering the newly reported ZP values. Additionally, this underscores how the presence of chemical groups that generate or possess partial charge densities in their structures can contribute to attractive interactions that outweigh repulsive forces.

PVA in the presence of RPMI also exhibited a decrease in ZP to −7.606 ± 0.648 mV, which was evidenced by a favored interaction. The pH-dependent variations in the ZP of KL and PVA+KL in RPMI medium were lower those observed for water-dispersed particles. It is a precious parameter because RPMI was the base for cytotoxicity assays. Similar were observed to KL-pH 2, KL-pH 5, and KL-pH 8 dispersed in RPMI, i.e., the ZP values of −12.325 ± 1.349 mV, −14.645 ± 0.568 mV, and −14.636 ± 0.286 mV, respectively, expressed a favored interaction. Finally, the mixtures of PVA+KL-pH 2, PVA+KL-pH 5, and PVA+KL-pH 8 dispersed in RPMI also had ZP values that remained within the range of +30 mV, evidencing attractive interactions. The importance of these results will be better understood in the cytotoxicity assays. Table 3 reveals that all samples showed stability during Zeta Potential analyses, despite the analysis involving a short measurement time. If the colloidal system is unstable, agglomerations will occur quickly, and the values will be much more discrepant than those obtained.

### 3.5 Cytocompatibility of PVA/Lignin Nanocomposites Assessed in Vero Cells

Vero cells are widely used for cytotoxicity testing, as they are well-established, easy to culture, and serve as a model for testing microbiological effects and vaccine production. Thus, the ability of previously characterized nanocomposites [PVA, PVA+ (LIGNIN pH 2, LIGNIN pH 5, LIGNIN pH 8)] to reduce the viability of Vero cells was evaluated. Treatment with the composites for 24 h did not result in a significant decrease in cell viability for any of the polymers tested (Figure 4).

**Figure 4:**
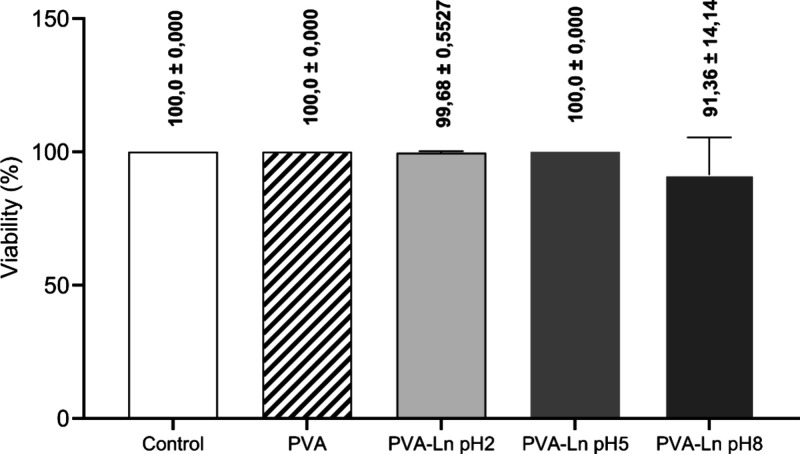
Graphical representation of the cell viability of Vero cells incubated for 24 h with polymers at different pH levels and compositions, evaluated by the colorimetric assay with resazurin. Absorbance values were normalized relative to the cell control (CC). Columns represent the mean and, rows represent the standard deviation; the numerical value above each column represents the mean ± standard deviation.

The composites did not exhibit significant cytotoxicity against VERO cells, indicating that, under these conditions, the polymers were safe. However, although lignin has beneficial antioxidant properties and potential selective cytotoxic effects, these effects vary depending on the type of lignin, its concentration, and the cell line evaluated. Certain lignin fractions, such as Kraft lignin from hardwood, have demonstrated the ability to induce intracellular production of reactive oxygen species (ROS) and cause oxidative DNA damage in HepG2 cells, despite exhibiting antioxidant properties in chemical assays.^38^ This suggests that, although lignin can act as an antioxidant, it can also induce oxidative stress under specific conditions.

Another study conducted by Siddiqui et al. demonstrated that the incorporation of drugs into nanoparticles resulted in a reduction in IC50 values for certain molecules, an effect observed in three distinct cell lines: HEK-293 (nontumor renal cells), A549 (lung tumor cells), and MCF-7 (breast tumor cells). A cytotoxic effect was observed in HEK-293 cells, highlighting the previously mentioned concern regarding the variability of cytotoxic responses depending on the cell line, lignin concentration, and its mode of application.^39^

While promising for applications in healthcare and drug delivery, comprehensive toxicological assessments are essential to ensure its safe use. Further studies are needed to fully understand the conditions under which lignin can become toxic to cells.

### 3.6 Immunomodulatory Effects of PVA/Lignin Nanocomposites on Cytokine Production

Cytokines are the master regulators of human immunity, and understanding whether any biomaterial is capable of being used for biomedical applications involves evaluating whether it modulates the production of these small peptides. To evaluate the ability of different polymers to modulate the immune response, we measured the levels of three cytokines involved in inflammatory and anti-inflammatory processes: IL-1β, IL-10, and TNF-α, all of which are associated with the innate immune response. Regarding IL-1β production, it was observed that PVA alone significantly stimulated its production compared to the control group, and the lignins adjusted to pH 2 and pH 8. Lignin at pH 5 exhibited a modulation pattern like that of PVA (Figure 5A). For IL-10, no statistically significant differences were detected among the tested groups (Figure 5B). However, pure PVA exhibited the lowest production of this cytokine (Figure 5B). Finally, TNF-α production was significantly increased in the group treated with PVA conjugated with lignin at pH 5 compared to the control and other treatments (Figure 5C).

**Figure 5:**
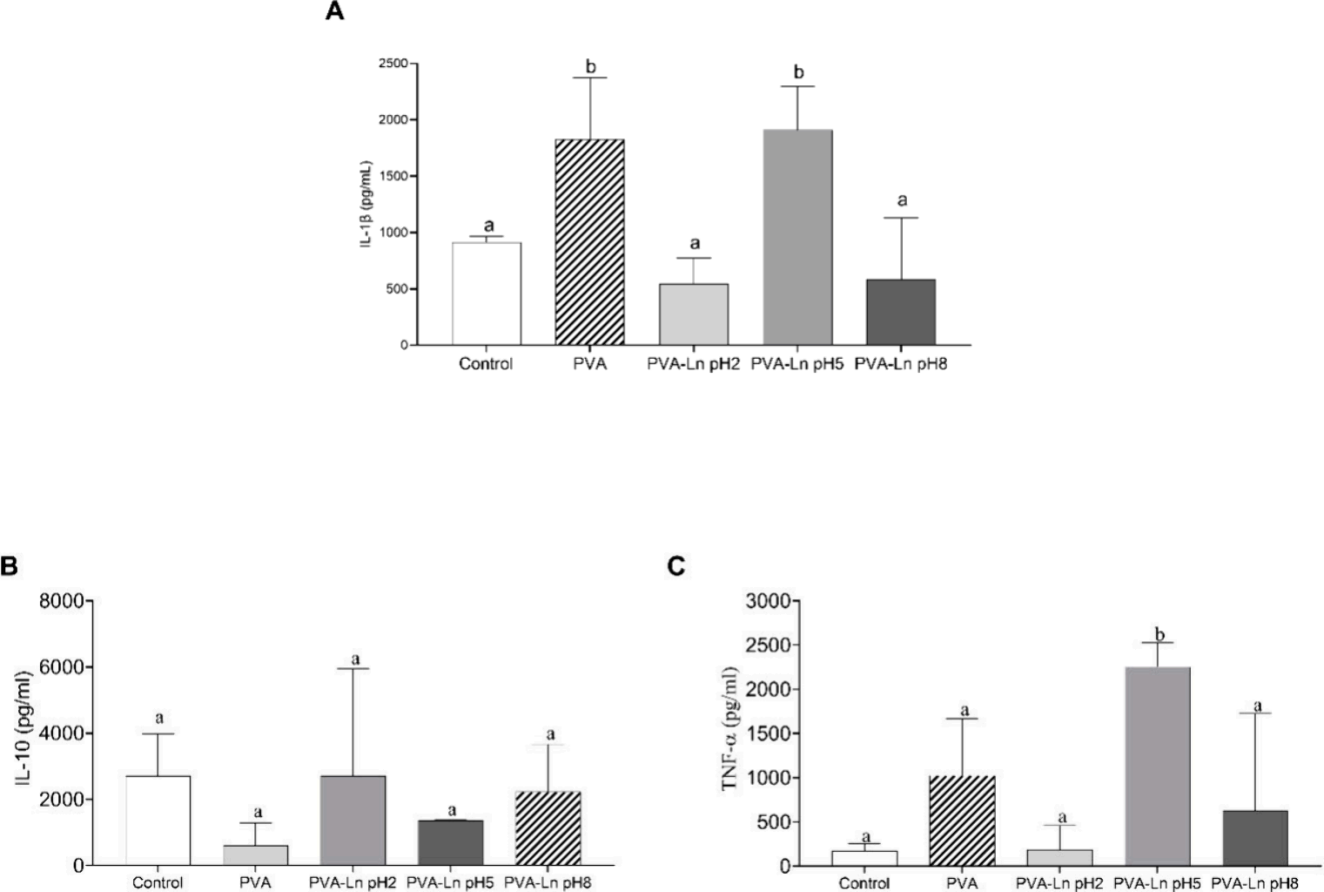
Evaluation of cytokine production in PBMCs treated with different polymers. (A) IL-1β, (B) IL-10, and (C) TNF-α. Results are expressed in pg/mL. Statistical analysis was performed using one-way ANOVA followed by the Holm-Šídák multiple comparisons test. Columns represent the mean, and rows represent the standard error of the mean. Significant differences were set at *p* < 0.05. Different letters indicate significant differences between the respective groups

Several studies report the modulation of the immune response by different types and concentrations of lignin. Splenocytes treated with lignin at 6 μg/mL for 24 or 48 h exhibited an altered pattern in TNF, IL-6, and IL-10 production, with a significant increase in these cytokines at both incubation times. This increase was accompanied by a reduction in NO production, which may be associated with decreased IFN-γ synthesis. The evaluated lignin was extracted from plants of the Opuntia species.^40^

Similarly, lignin isolated from Conocarpus erectus leaves stimulated the release of cytokines involved in the Th1 immune response.^41^ Additionally, immune response modulation in bovine cells has been demonstrated with hydrolyzed lignin from Pinus taeda, which induced a reduction in TNF-α production and an increase in IL-8 secretion, suggesting monocyte activation and a potential antioxidant response.^42^

Studies using lignin nanoparticles (LigNPs) in zebrafish models have demonstrated increased cytokine recruitment, aiding in inflammation resolution and tissue regeneration.^43^ One of the primary mechanisms of lignin action appears to be related to lymphocyte activation and cytokine synthesis induction, mediated by interactions between antigen-presenting cells (APCs) and T cells, in which IL-2 plays a central role in amplifying the immune response.^44,45^

In agreement with our findings, lignin and its derivatives demonstrate significant potential in modulating cytokine production, primarily through immune cell activation and APC-T cell interactions. These discoveries reinforce lignin’s potential as a therapeutic agent for immune response regulation and inflammation control.

## 4 Conclusions

Kraft lignin fractionation by precipitation at different pH values significantly influenced the physicochemical, morphological, and biological properties of electrospun poly­(vinyl alcohol) (PVA) composites. GPC and FTIR analyses demonstrated that pH modulation directly affected lignin molar mass distribution and functional group composition, which in turn governed dispersion behavior, surface charge, and fiber morphology. Zeta potential and particle size measurements revealed distinct pH-dependent colloidal stability profiles, highlighting the fraction obtained at pH 5 as exhibiting a favorable balance between surface charge stability and morphological characteristics.

Importantly, all PVA/lignin systems showed good cytocompatibility in Vero cells, with no significant reduction in cell viability after 24 h of exposure. In addition, the composites displayed differential immunomodulatory behavior, as evidenced by selective modulation of IL-1β, IL-10, and TNF-α production. Notably, the PVA–lignin system fractionated at pH 5 promoted a significant increase in TNF-α levels, indicating a controlled activation of innate immune response without compromising cell viability. Overall, these findings demonstrate that pH-controlled fractionation of Kraft lignin is an effective strategy to tailor the structural, colloidal, and immunological properties of electrospun PVA-based biomaterials, supporting their potential application in biomedical systems where immune interaction and biocompatibility are critical.

## Supplementary Material



## Data Availability

All data used in this manuscript are available in the Supporting Information.

## Notes

Schematic illustration of the experimental workflow and conceptual framework underlying the development and biological evaluation of electrospun nanofibrous biomaterials based on fractionated kraft lignin at different pH conditions; graphical representation of lignin fractionation, electrospinning processing, and subsequent biocompatibility and immunomodulatory assessment in mammalian cell models (Vero cells and PBMCs); overview diagram summarizing the relationship between material preparation parameters and biological response trends relevant to candidate biomaterial selection for future immunomodulatory applications (PDF)
