# Supplementary material for: Electrospun Poly(vinyl alcohol) Composites Containing pH-Fractionated Kraft Lignin: Structural Characterization, Biocompatibility, and Immunomodulation
Source: ACS Omega. 2026 Mar 5;11(10):15858–68. doi: 10.1021/acsomega.5c09388 (PMC13000596; doi:10.1021/acsomega.5c09388)
Supplement: Supplementary file 1 [file ao5c09388_si_001.pdf]

Supporting Information - Electrospun polyvinyl alcohol composites containing pH-fractionated kraft lignin: structural characterization, biocompatibility and, immunomodulation

Chamberttan Souza Desidério [1]*, Hugo Felix Perini[2], Beatriz Sodré Matos[2], Antônio Aprigio da Silva Curvelo[3], Marcos Vinicius da Silva[2], Carlo José Freire Oliveira [1], Luís Carlos de Morais[4]

[1]Laboratory of Immunology and Omic Sciences - Department of Microbiology, Immunology, and Parasitology, Institute of Biological and Natural Sciences, Federal University of Triângulo Mineiro – UFTM, Uberaba, MG, Brazil

[2]Parasitology and Molecular Biology Research Laboratory - Department of Microbiology, Immunology, and Parasitology, Institute of Biological and Natural Sciences, Federal University of Triângulo Mineiro – UFTM, Uberaba, MG, Brazil

[3]Department of Physical Chemistry, Organic Physical Chemistry Group, São Carlos Chemistry Institute, University of São Paulo - USP.

[4]Polymer research and technological application laboratory – LaPPAT - Institute of Exact, Natural Sciences, and Education (ICENE), Federal University of Triângulo Mineiro – UFTM, Uberaba, MG, Brazil.

Figure S.1 – Workflow Manuscript

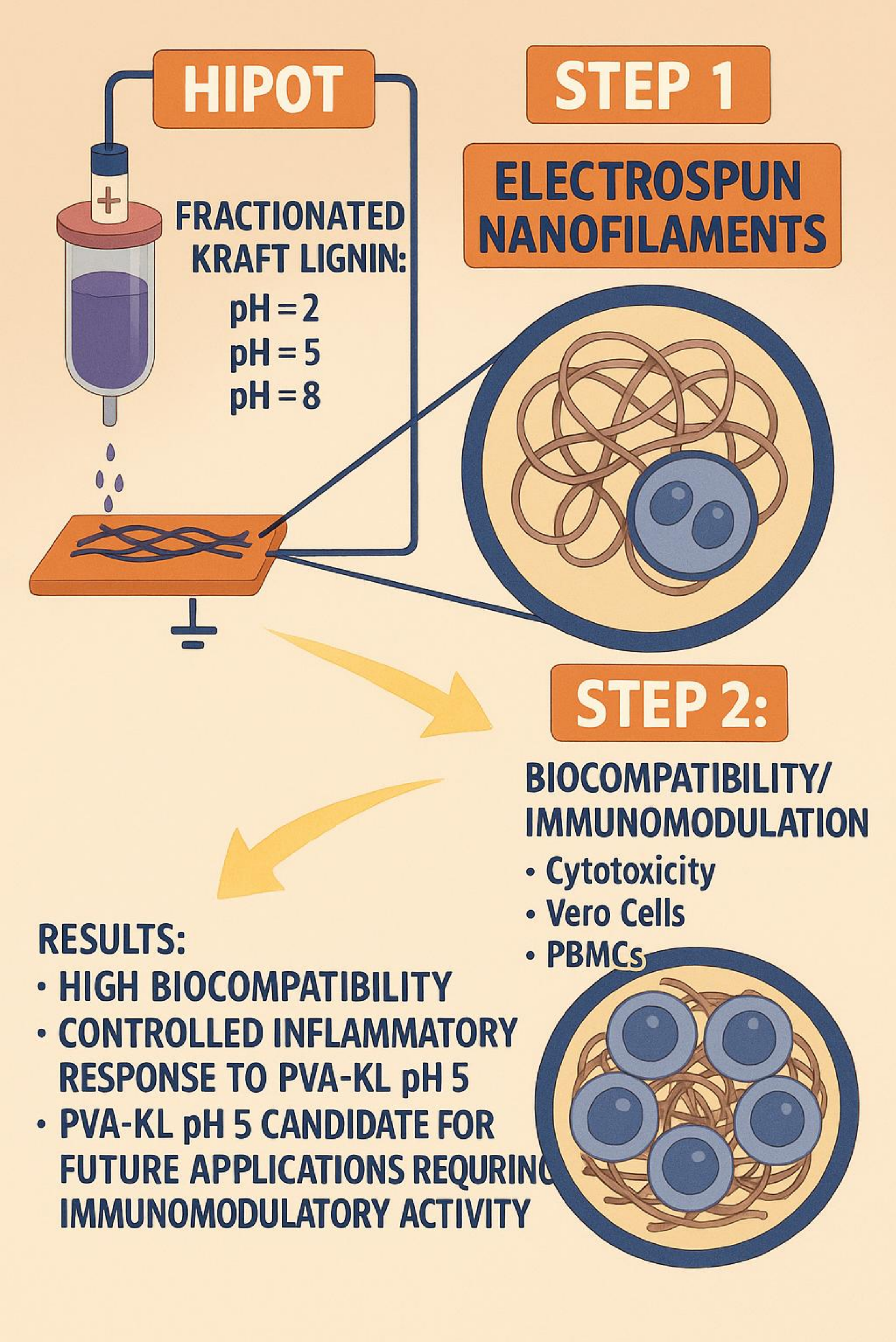

Figure S.1. The structure of our study was divided into three main parts: (A) Preparation of

PVA dispersions mixed with fractionated lignins at different pH levels (2, 5, and 8) followed by electrospinning of the mixtures. (B) Electrospun nanofibers in contact with cells (Vero cells, PBMCs) followed by biocompatibility/immunomodulation assays. (C) Obtained results and data analysis.
